# Supplementary material for: Zooplankton variability in the Strait of Georgia, Canada, and relationships with the marine survivals of Chinook and Coho salmon
Source: PLoS One. 2021 Jan 25;16(1):e0245941. doi: 10.1371/journal.pone.0245941 (PMC7834739; doi:10.1371/journal.pone.0245941)
Supplement: S1 Fig — Baseline (climatology) period was 1996–2010. Y-axes are the annual anomalies; note the scales differ. Y-axes are labelled with the abbreviated names of each zooplankton group (abbreviated names are defined in Table 1). (PDF) [file pone.0245941.s004.pdf]

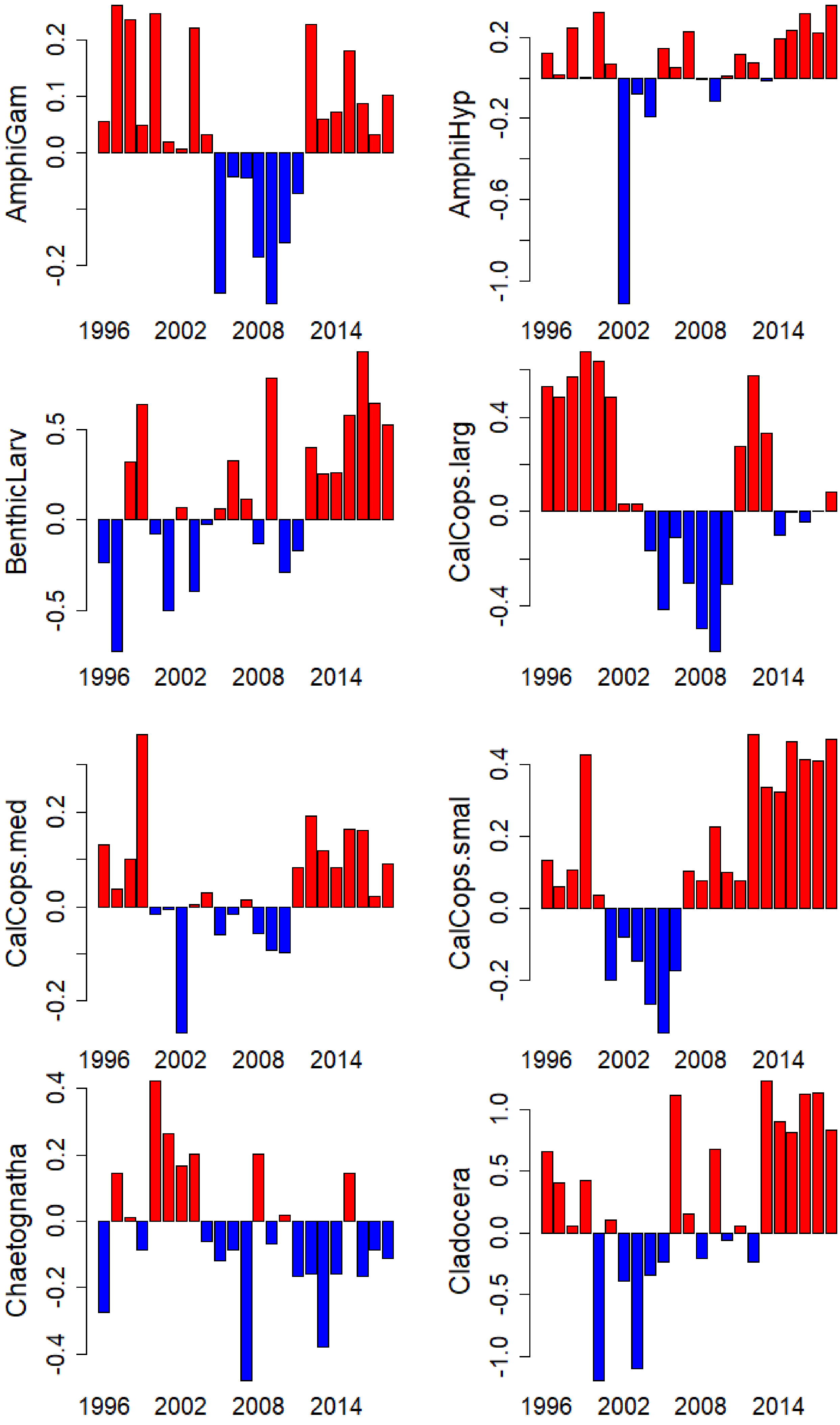
AmphiGam
AmphiHyp
BenthicLarv
CalCops.larg
CalCops.med
CalCops.smal
Chaetognatha
Cladocera
1996
2002
2008
2014

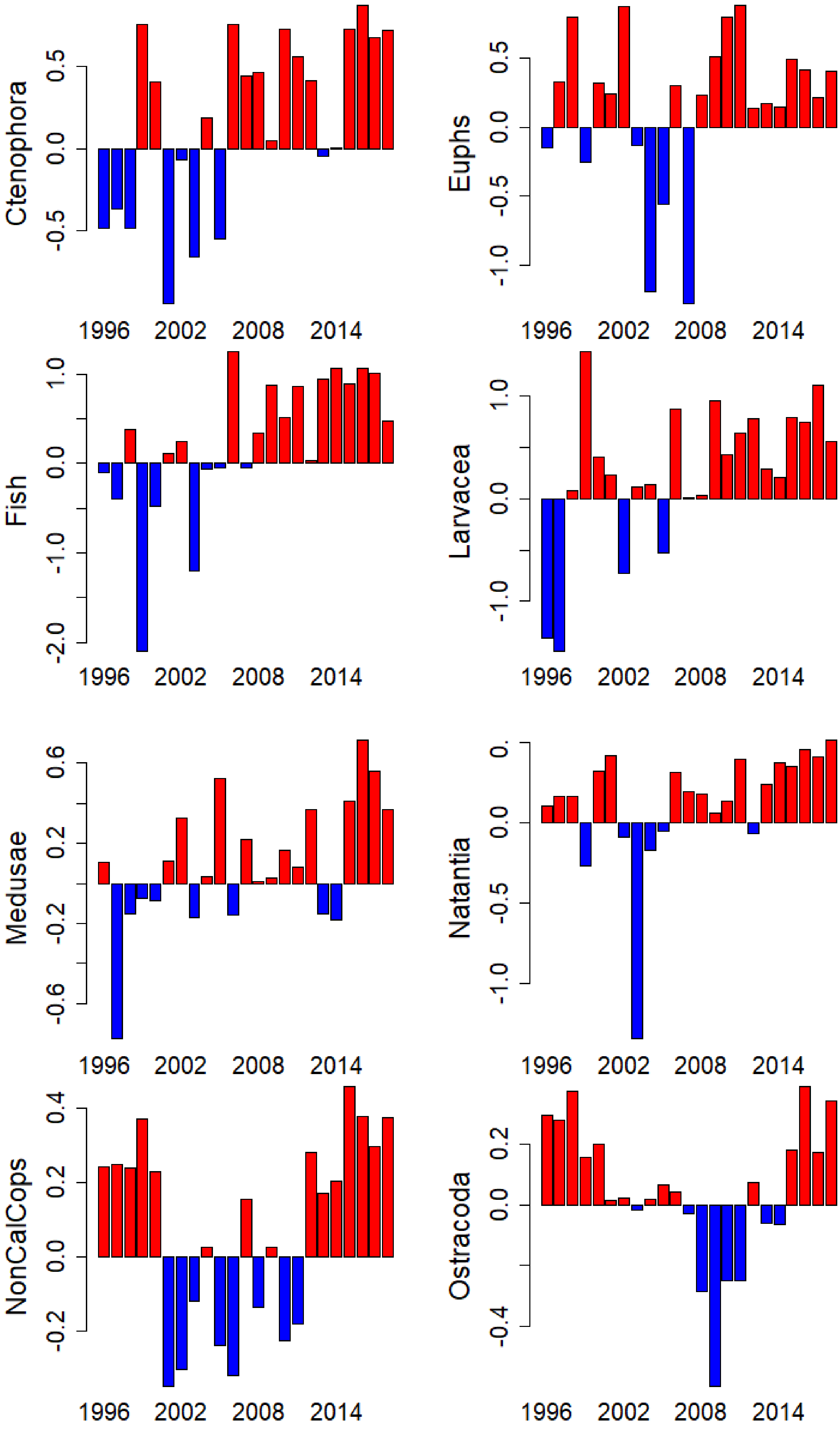

Ctenophora
Euphs
Fish
Larvacea
Medusae
Natantia
NonCalCops
Ostracoda
1996
2002
2008
2014

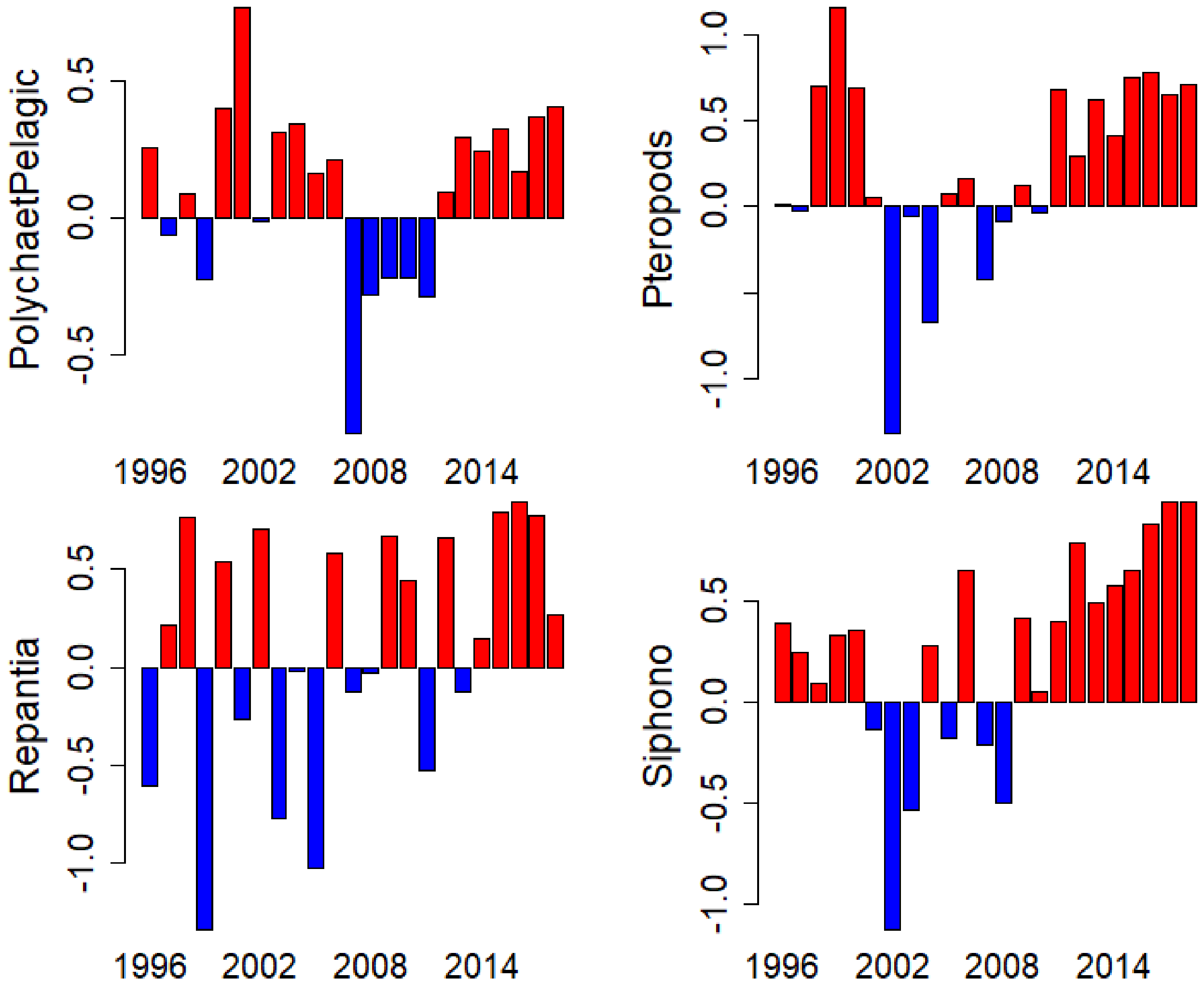

S1 Fig. Annual anomalies of $\log_{10}$ biomass (g m^{-2}) of the 20 zooplankton taxonomic groups, from 1996 to 2018. Baseline (climatology) period was 1996-2010. Y-axes are the annual anomalies; note the scales differ. Y-axes are labelled with the abbreviated names of each zooplankton group (abbreviated names are defined in Table 1).
