# Supplementary material for: Zooplankton variability in the Strait of Georgia, Canada, and relationships with the marine survivals of Chinook and Coho salmon
Source: PLoS One. 2021 Jan 25;16(1):e0245941. doi: 10.1371/journal.pone.0245941 (PMC7834739; doi:10.1371/journal.pone.0245941)
Supplement: S2 Fig — (A) Cowichan River Chinook, (B) Harrison River Chinook, (C) Puntledge River Chinook, and (D) Big Qualicum Coho salmon populations for ocean entry years 1996 to 2015 for Chinook, and to 2017 for Coho. Black dots and lines represent the observed early (for Chinook) or total (for Coho) marine survivals. Blue line and shading represent 95% confidence bands about a loess smoother applied to these annual values (derived from a general additive model with year as the independent variable) to better show the general patterns. (PDF) [file pone.0245941.s005.pdf]

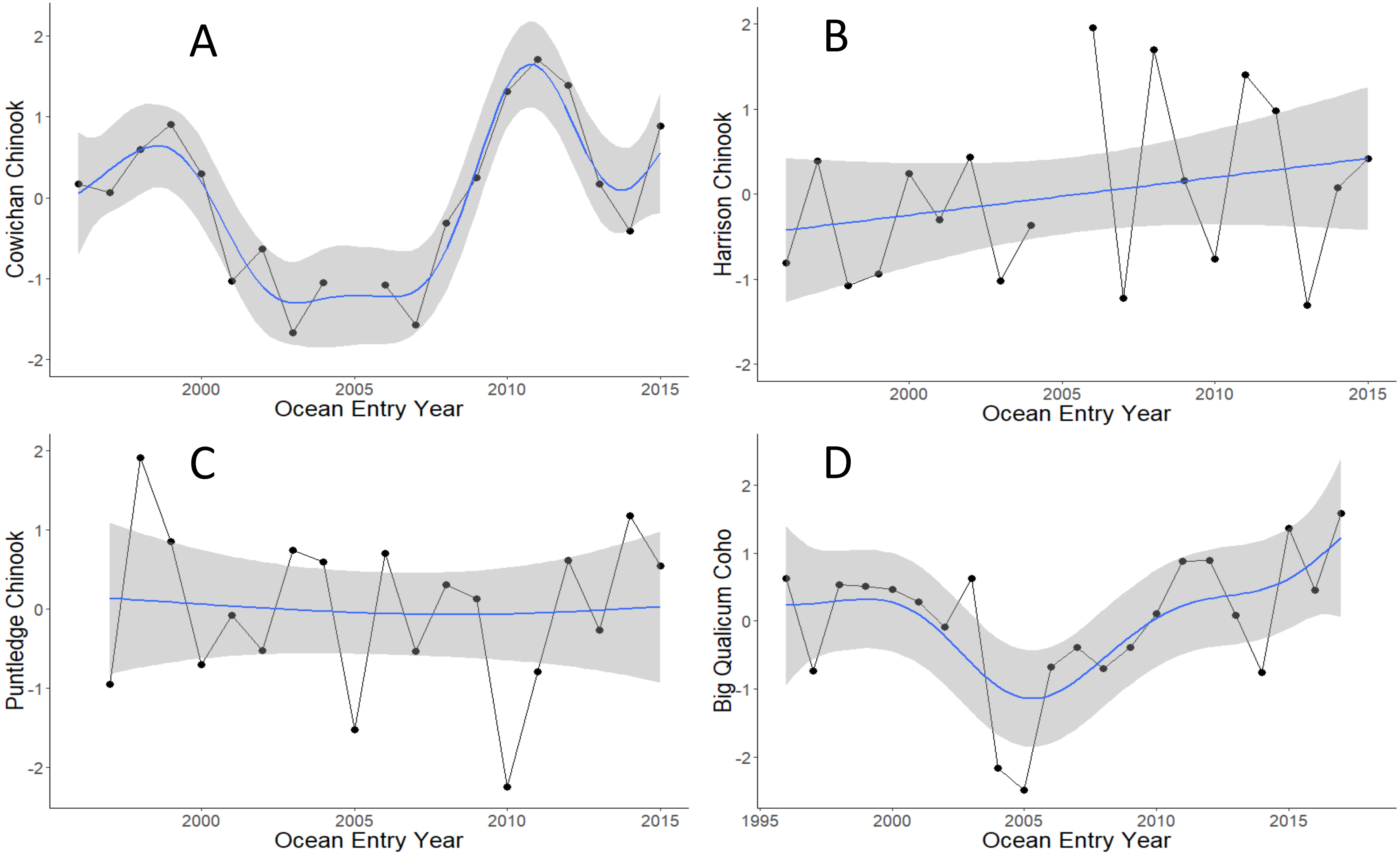

S2 Fig. Transformed and scaled salmon early marine survival patterns. (A) Cowichan River Chinook, (B) Harrison River Chinook, (C) Puntledge River Chinook, and (D) Big Qualicum Coho salmon populations for ocean entry years 1996 to 2015 for Chinook, and to 2017 for Coho. Black dots and lines represent the observed early (for Chinook) or total (for Coho) marine survivals. Blue line and shading represent 95% confidence bands about a loess smoother applied to these annual values (derived from a general additive model with year as the independent variable) to better show the general patterns.
