# Supplementary material for: Zooplankton variability in the Strait of Georgia, Canada, and relationships with the marine survivals of Chinook and Coho salmon
Source: PLoS One. 2021 Jan 25;16(1):e0245941. doi: 10.1371/journal.pone.0245941 (PMC7834739; doi:10.1371/journal.pone.0245941)
Supplement: S1 File — (PDF) [file pone.0245941.s006.pdf]

S1_File. Numerical simulation to examine the effect of uneven sampling among years.

In this study, the sampling effort each year from 1996 to 2018 was very uneven, ranging from a low of 4 in 1996 and 2000 to a high of 156 in 2016 (Table 3). The average number of samples each year during the best sampled period (2015 to 2018) was 112, whereas the average number of samples during the poorest sampled period (2003 to 2007) was 12. It is possible, therefore, that the total zooplankton biomass anomalies from 2015 to 2018 may have been influenced by the large number of samples in those years. Conversely, it is possible that the low total zooplankton biomass anomalies from 2003 to 2007 were due to the low number of samples in those years.

We conducted a numerical simulation, using the available total zooplankton biomass data, to assess these two questions.

1) Could the higher sampling effort in 2015 to 2018 have produced larger total zooplankton biomass anomalies for those years – i.e. what might the distribution of annual anomalies look like if only 12 samples had been collected in each of these recent years?

To examine this question we simulated what the annual biomass anomalies might have been if only 12 samples had been collected in each of those years, based on the observed samples. We randomly took 12 samples (without replacement) from the available distribution of samples each year, and calculated their annual biomass anomalies. We repeated this process 1000 times to generate a distribution of possible biomass anomalies based on only 12 samples each year. The results (Fig. A, below) show that the anomalies calculated using the full data set (i.e. with an average of 112 samples each year) are almost identical to the mean anomalies that would likely have been observed if only 12 samples had been collected each year. We conclude that it is unlikely that the higher anomalies in 2015 to 2018 occurred because of the larger number of samples in those years, compared with the number of samples collected from 2003 to 2007 (average of 12 samples a year).

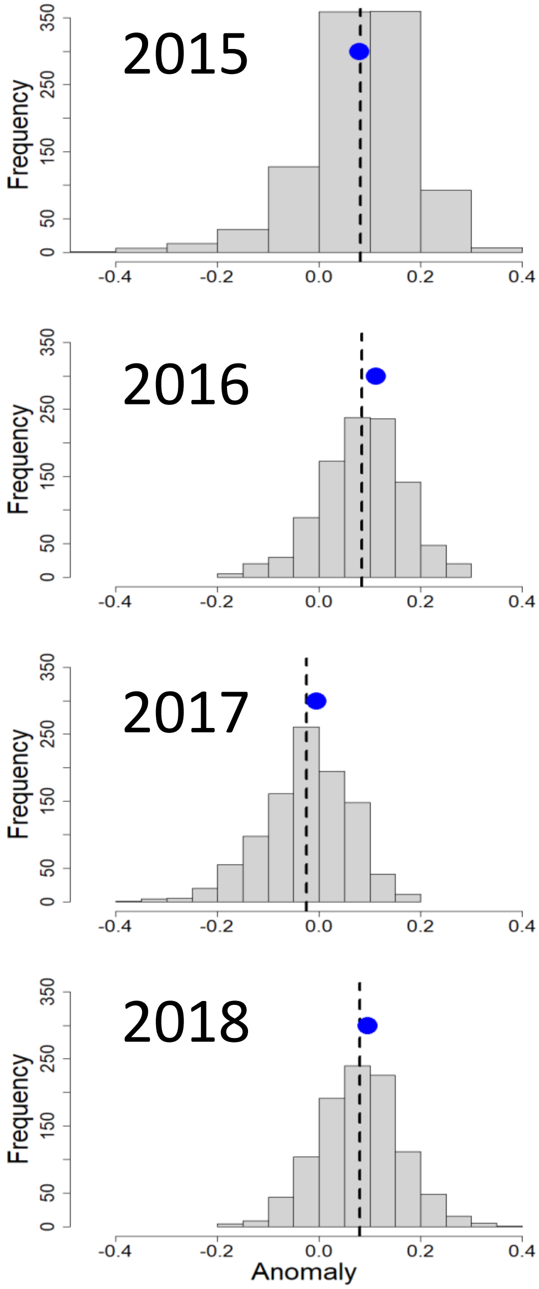

Fig. A. Histograms of total zooplankton biomass anomalies calculated from 12 samples drawn randomly (without replacement) from the observed samples in each year and repeated 1000 times. The vertical dashed line shows the mean biomass anomaly of the 12 random samples; the blue dot shows the annual anomaly of total zooplankton biomass for each year calculated using the full data set. Climatology period for all simulations was 1996 – 2010 (as with the original analysis).

2) What is the probability that the sequence of low total zooplankton biomass anomalies in 2003 to 2007 (5 years in a row) would have occurred if the true distribution of samples was similar to 2015 to 2018 (i.e. with true annual anomalies near or somewhat above the climatological mean, 1996-2010) and 12 samples had been collected each year?

The highest annual anomaly of total zooplankton biomass for the period 2003 to 2007 was -0.09 (in 2006, e.g. Fig. 2D). From the simulation above, anomalies less than or equal to -0.10 occurred in 5.4% of the distribution for 2015, in 2.5% of the distribution for 2016, in 18.3% of the distribution for 2017, and in 1.3% of the distribution for 2018. If the true distribution of total zooplankton biomass in the period 2003 to 2007 was the same as that from 2015 to 2018, then the probability of getting an anomaly in the lowest 20% of the distribution for each year, for five years in a row, would be: 0.2 * 0.2 * 0.2 * 0.2 * 0.2 = 0.00032. This indicates that the probability of getting five biomass anomalies with values less than -0.10 in a row, if the true distribution of total zooplankton biomass was as observed in 2015 to 2018 (i.e. with annual anomalies at or above the climatological mean) is unlikely. In addition, Fig. B (below) shows that the seasonal mean total zooplankton biomass tended to be lower in all seasons during 2003 to 2007, suggesting these low biomass values were not due to one season alone. We conclude, therefore, that the total zooplankton biomass, and therefore the biomass anomalies, in 2003 to 2007 was lower than the climatological mean, even though an average of only 12 samples were collected each year.

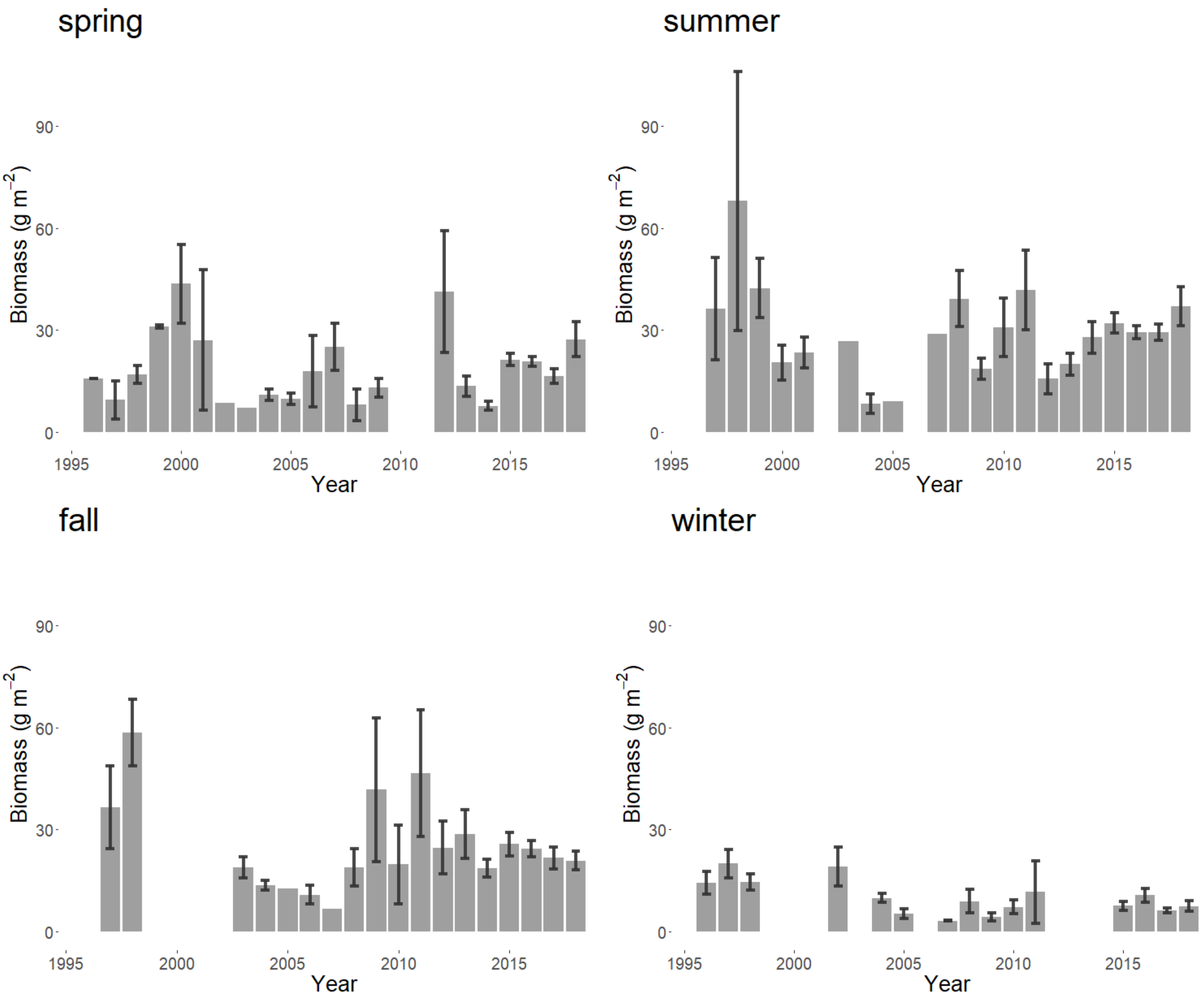

Fig. B. Total zooplankton biomass (g m^{-2}) by season. The vertical black lines represent one standard error about the mean seasonal value for each year.
